# Supplementary figures and images for: Delayed Surgical Management of Congenital Syndactyly Improves Range of Motion: A Long-Term Follow-Up
Source: J Clin Med. 2025 May 5;14(9):3200. doi: 10.3390/jcm14093200 (PMC12072538; doi:10.3390/jcm14093200)

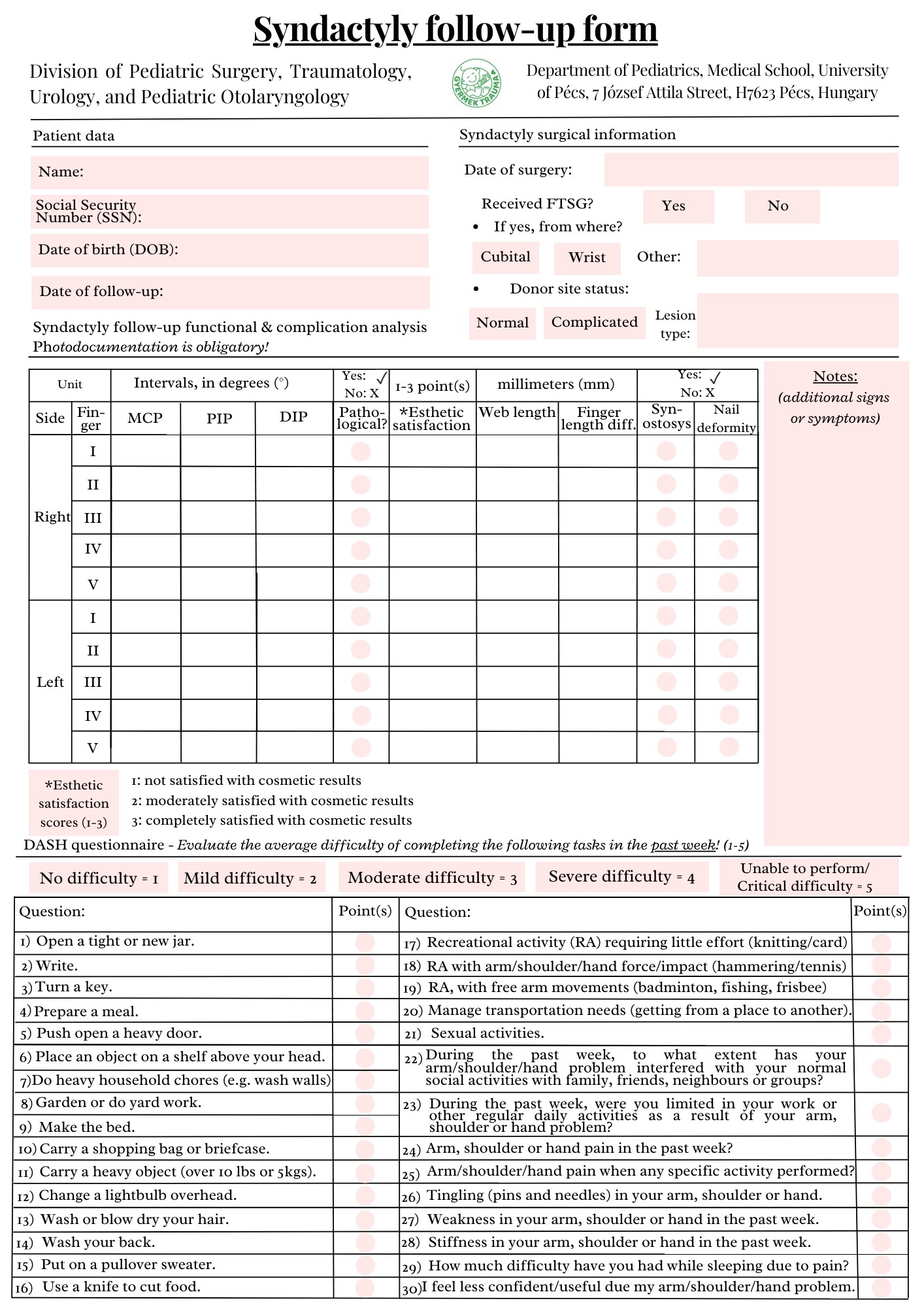

Supplement: Supplementary file 1 [file jcm-14-03200-s001.zip › SFig1.jpg]
